# Health Related Quality of life Amongst Refugees: A meta Analysis of Studies Using the SF-36

**DOI:** 10.1007/s10903-024-01615-4

**Published:** 2024-07-03

**Authors:** Ryan Essex, Poonkulali Govintharjah, Rita Issa, Erika Kalocsányiová, Dostin Lakika, Marianne Markowski, James Smith, Trevor Thompson

**Affiliations:** 1 Institute for Lifecourse Development, The University of Greenwich, Old Royal Naval College, Park Row, London, SE10 9LS UK; 2 University of Western Sydney, Sydney, Australia; 3 University College London, London, England; 4 University of East Anglia, Norwich, England; 5 African Centre for Migration & Society, Wits University and French Institute of South Africa, Johannesburg, South Africa

**Keywords:** Systematic Review, Meta-analysis, Refugees, Asylum seekers, SF-36, Health-related quality of life

## Abstract

The health of refugees has been widely documented, as has the impact of a range of factors throughout the migration journey from being exposed to violence to the impacts of immigration detention. This study adds to our understanding of health-related quality of life amongst refugees and asylum seekers by evaluating health-related quality of life as measured by the Short-Form 36 (SF-36) Health Survey using meta-analysis. The aims of this study were to (1) provide a summary and overview of health-related quality of life (as measured by the SF-36), including the extent to which this varies and (2) explore the factors that influence health-related quality of life (as measured by the SF-36) amongst refugees and asylum seekers. A search was undertaken of MEDLINE, CINAHL, PSYCINFO and SCOPUS, returning 3965 results. Papers were included if they sampled refugees (or asylum seeker or those with refugee-like experiences) and used the SF-36 (or its variants) as an outcome measure. Mean scores and standard deviations were pooled using a random effects model. The pooled sample size was 18,418. The pooled mean scores for the SF-36 physical summary measures was 54.99 (95% CI 46.01–63.99), while the mental health summary measure was 52.39 (95% CI 43.35–61.43). The pooled mean scores for each of the sub-scales ranged from 49.6 (vitality) to 65.54 (physical functioning). High heterogeneity was found between both summary measures and all sub-scales. In comparison to SF-36 results from general populations in high and middle income countries, these results suggest that refugee quality of life is generally poorer. However, this varied substantially between studies. One issue that is not well clarified by this review are the factors that contributed to health-related quality of life.

**Supplementary Information:**

The online version contains supplementary material available at 10.1007/s10903-024-01615-4.

## Introduction

In 2022 there were over 100 million people who were forcibly displaced globally [1]. Amongst these were over 40 million refugees and asylum seekers, those who have fled their country and sought (or are seeking, as is the case with asylum seekers) protection in another country because of violence or persecution. In addition to having to flee their home countries, the vast majority of refugees and asylum seekers are subjected to significant hardships. Violence and persecution prompt many to flee their home countries. While seeking safety, many live in unsafe conditions, most are in temporary camps for protracted periods of time. The precarity of the conditions in these camps has been well documented, with little food, water and sanitation, with violence an ongoing threat [2]. Many find themselves on perilous journeys, by land and sea. The Mediterranean, for example, has seen over 1.7 million attempted crossings since 2016. This has resulted in almost 30,000 people reported dead or missing [3]. For those who make it to a country where protection could be provided, they often face further hardship in the form of detention or persecutory immigration policies, which leave many destitute and waiting years for a decision on their claims for asylum [4]. Even when asylum is granted, many post-migration challenges remain; financial strain and/or unemployment, housing difficulties, language problems, weak social networks and discrimination.

The health of migrants and more specifically, refugees and asylum seekers has been widely discussed, as has the impact of the migration journey on health and wellbeing. Several meta-analyses now exist that document the impact of the migration journey on mental heath. Together these studies suggest that anxiety, depression and post-traumatic stress disorder are far more prevalent amongst refugees and asylum seekers in comparison to the general population [5]. While there is a growing body of work in this area on mental health, health-related quality of life has received relatively less attention. Some recent exceptions include a systematic review carried out by Gagliardi, Brettschneider [6] that explored health-related quality of life as measured by the WHOQOL-Bref instrument. While this study did not utilise meta-analysis, the authors found that scores were generally lower than population norms, however also had high heterogeneity. Similarly, a systematic review by van der Boor, Amos [7] examined the factors associated with quality of life for asylum seekers and refugees in high-income countries, finding that quality of life was largely associated with social and mental-health related factors.

Given the nature of these populations, the adverse experiences they often face and ongoing challenges in resettlement, the need for further clarity in relation to health-related quality of life is pressing. This study seeks to add to our understanding of health-related quality of life amongst refugees and asylum seekers by evaluating the Short-Form 36 (SF-36; 8) using meta-analysis.

The aims of this study were to (1) provide a summary and overview of health-related quality of life (as measured by the SF-36), including the extent to which this varies and (2) explore the factors that influence overall and specific components of quality of life as measured by the SF-36 amongst refugees and asylum seekers.

## Methods

A systematic review was carried out to identify all relevant studies; those that utilised the SF-36 (and its variants). PRISMA guidelines [8] were followed and a study protocol was registered with PROSPERO (CRD42023465533). The search strategy, screening, data extraction and analysis are outlined below.

### Search Strategy

A search was carried out on 20/09/2023 utilising the following databases: PSYCINFO, CINAHL, MEDLINE and SCOPUS (which incorporates both MEDLINE and EMBASE). Search terms were developed to capture the core concepts, related to the population and outcome of interest. The final search terms were: refugee OR “asylum seeker” AND “self-reported health” OR “self reported health” OR “self-perceived health” OR “subjective health” OR “physical health” OR “health status” OR “quality of life” OR QoL OR “short form” OR sf-36 OR sf-20 OR sf-12 OR sf-6.We carried out a further manual search of references lists of studies found to be eligible.

### Eligibility Criteria

No time or language limits were placed on studies. Studies were included if the sample included refugees or asylum seekers. We included studies that self-identified their samples as refugees and asylum seekers. Where studies included a mixed sample (for example migrants and refugees) we included these studies if the majority came from refugee/asylum seeker backgrounds or data specific to the refugees or asylum seekers could be extracted. We also included studies where samples were likely to have had refugee-like experiences. For example, a sample of migrants who had escaped political persecution would be included even if the study did not explicitly identify them as refugees or asylum seekers. We included studies that sampled refugees and asylum seekers in all settings, this included those in transit, those who had resettled and those in clinical (i.e., hospital or medical) settings.

Studies were included if they used the SF-36 (or any of its variants) as an outcomes measure. The SF-36 is a self-report measure of health and wellbeing and was designed to give an overall picture of general health status. For ease and unless specified, we will refer to all variations of this questionnaire (see below) as the SF-36. The SF-36 has been translated into more than 40 languages and used in thousands of studies with a range of general and clinical groups. The SF-36 consists of two summary measures – physical and mental health and eight scales: physical functioning (10 items), a scale which includes questions related to everyday physical activities; role-physical (4 items), a scale which includes questions related to the physical demands of work and other daily activities; bodily pain (2 items), a scale that explores pain and the extent to which it interferes with daily activities; general health (5 items), a scale which explore general perceptions of health and future health outlook; vitality (4 items), a scale exoring levels of energy and fatigue; social functioning (2 items), a scale measuring social functioning; role-emotional (3 items), a scale exploring the extent to which emotional problems interfere with work and daily activities and mental health (5 items), a measure of overall psychological wellbeing. Scales are standardised and scored from 0 to 100, with higher scores indicating better health.

There are several variations of the SF-36, including the SF-8 [9], SF-12 [10] (which only include the physical and mental health summary measures) and the SF-20 (which only includes the six of the above scales) [11]. In addition to there being several versions that differ in length, there is a RAND [12] and Medical Outcomes Study [13] version, each of which contain the same items, but are scored slightly differently. We opted to include all versions of the SF-36 as generally different versions have been found to be highly correlated [14]. We also planned to explore the impact that the choice of instrument had on scores in our sensitivity analysis (see below). Studies had to contain extractable data for at least one of the sub-scales or summary measures. Only cross-sectional studies were included.

### Screening and data Extraction

Screening was undertaken in two phases. A first screen was carried out by RE examining the titles and abstracts of articles. A second full text screen was carried out by RE and EK. Each paper was screened independently, by at least two authors. Disagreements were resolved with a discussion with the team. RE, EK and MM contributed to data extraction. Data was extracted independently by at least two authors, except on the two papers which were in German, where MM carried out the data extraction. SF-36 scores, along with demographic and study characteristics were extracted, including the sample size, country where the study took place, the type of outcomes measure used, the mean age of the sample, whether the sample came from the general population or was clinical and whether the sample was residing in camps or an urban setting.

### Quality Appraisal

We opted to use the JBI’s critical appraisal tool for cross sectional studies (https://jbi.global/critical-appraisal-tools). Each study was independently rated by at least two authors. Disagreements were resolved with a discussion to reach a final, collated score.

### Publication bias

Potential publication bias was explored utilising funnel plots (visualising effect sizes against SEs), where asymmetry can indicate possible bias, and with Egger’s test with *p* < .10 indicating possible publication bias.

### Data Transformation and Analysis

While a number of studies were excluded because no data could be extracted, where possible data was transformed. This generally involved converting standard errors and confidence intervals. To do this we used the Cochrane RevMan calculator [15]. For a small number of studies we either pooled means and standard deviations (where two groups were compared for example) or extracted data for only one group in the study in question. The specific details for each study where this was done will be provided below.

Meta-analysis was used to systematically synthesize the findings of the studies retrieved from the search. Mean scores and standard deviations were pooled using a random effects model with tests for heterogeneity (see below). The meta package [16] in R [17] was used to carry out this analysis. Plots were created using ggplot [18].

### Analysis of sub-groups

While we had planned to carry out a formal analysis of sub-groups, we opted not to do this for a range of reasons which are outlined below (see results). Instead we carried out a more informal exploration of the data, to help in identifying patterns in the data and any study characteristics that appeared to influence this.

### Heterogeneity

The existence of heterogeneity was explored with Cochran’s Q statistic (where *p* < .05 indicates heterogeneity is present). The magnitude of the variation in effect sizes across studies with Higgin’s I2 statistic was also utilised. This statistic estimates the proportion of variance in effect sizes due to true heterogeneity (from 0 to 100%), with higher values representing greater inconsistency in effect size across studies. Finally, we also report τ as a measure of heterogeneity for each comparison, which gives the SD of the effect size estimates.

### Sensitivity Analysis

We also performed sensitivity analysis to assess how robust our findings were. We planned to removed outliers, that is, studies with unusually high or low SF-36 scores on any of the subscales or summary measures and studies which scored relatively low on the JBI quality appraisal instrument in question, for our purposes this meant not meeting at least 70% of the criteria on any given instrument. The results of this and the above search and analysis will be reported below.

### Patient and Public Involvement

Patients and the public were not involved in the development of this manuscript.

## Results

**Fig. 1:**
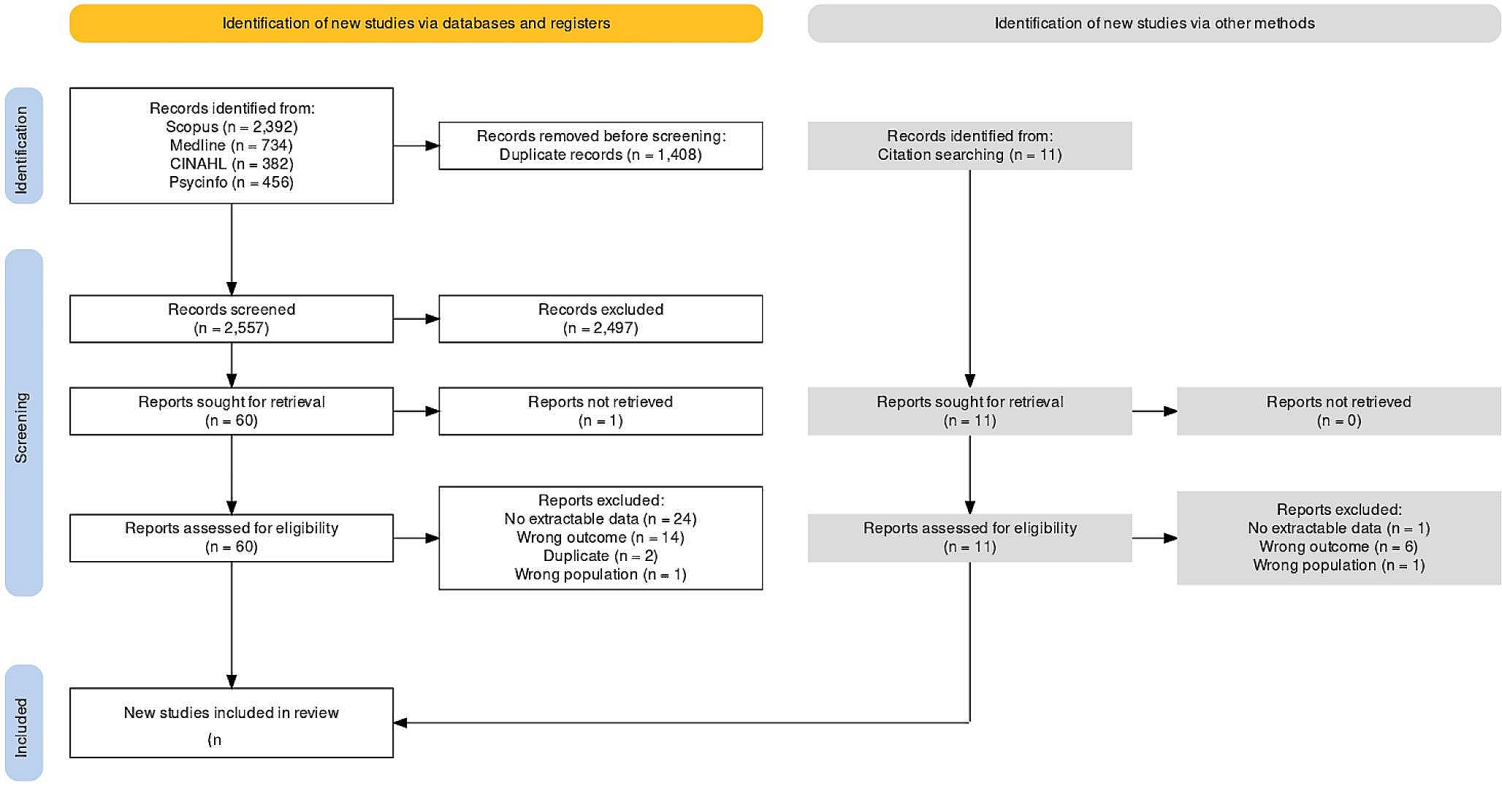
PRISMA flow diagram [40]

The search returned 3965 results. Results were imported into Rayyan [19] where duplicates were removed, leaving 2557 papers. A title and abstract screen left 61 articles, a full text screen was then carried out. A further 11 papers that were found in reference lists of included papers, which were also screened. After screening, 20 papers met the inclusion criteria [20–39]. The search results are summarised in a PRISMA diagram (Fig. 1).

The 20 studies included reported data from over two decades (1999–2023) and were carried out in Croatia [28], Germany [20, 21, 25], Greece [22, 23], Jordan [37], Kosovo [35, 36], Malaysia [34], Netherlands [30], South Sudan [38], Switzerland [32], Thailand [33], Uganda [39] and the US [24, 26, 27, 29, 31]. Studies were also categorised as high income countries (*n* = 13) and low and middle income countries (*n* = 7). Studies used the SF-36 (*n* = 11), SF-12 (*n* = 6), SF-8 (*n* = 2), SF-20 (*n* = 1). Two studies utilised the RAND version of the SF-36/12 [32, 37] while the remainder used the Medical Outcomes Study version(s). The majority of studies had either samples of refugees (*n* = 17), mixed samples (*n* = 2), while the remaining study had a sample of internally displaced people. Fourteen studies included outcomes from samples who lived in ‘urban’ environments, five who lived in ‘camps’ and one which included a mixed sample. The total sample size of these studies was 18,418. Not all studies reported individual component scores. The most commonly reported measures were the mental and physical health summary measures (n = 11 and n = 11 respectively).

For two studies with multiple assessments, pooled mean and standard deviations were used [20, 26]. Data was also selectively extracted from another three where we felt that data could not be pooled (as the groups being compared were too different). For the study carried out by Eisenman, Gelberg [24] we only extracted data for the group who experienced political violence (as opposed to the group who did not), for the study carried out by Eytan, Toscani [41], we extracted data from the group who had not been diagnosed with post-traumatic stress disorder (as opposed to those who were diagnosed) and for the study carried out by Wangmo [32] we extracted data for those who had resettled in Switzerland (as opposed to those resettled in India).

### SF-36 sub Scales

The pooled mean scores for each of the SF-36 subscales was calculated. Subscales ranged from 49.60 (vitality) to 65.54 (physical functioning). All subscales had significant (Q) and substantial (I^2^, τ^2^) heterogeneity. These results are summarised in Table 1, with forest plots included as supplementary material.

**Table 1:** Summary of results for SF-36 sub scales

Scale	Pooled mean	CI	Range	Q	I^2^	τ^2^
Physical functioning	65.54	56.98–74.09	43.43–81.70	2481.12 (*p* < .001)	99.6%	160.40
Physical role	56.55	45.39–67.70	19.19–80.60	1444.39 (*p* < .001)	99.3%	269.93
Pain	60.11	52.92–67.29	40.92–76.40	1633.49 (*p* < .001)	99.2%	125.74
General health	50.42	43.62–57.21	25.26–65.50	2264.76 (*p* < .001)	99.5%	112.48
Vitality	49.60	44.18–55.01	32.88–56.80	1212.16 (*p* < .001)	99.3%	56.09
Social functioning	61.66	53.03–70.28	43.01–81.51	1648.12 (*p* < .001)	99.4%	161.73
Emotional role	53.32	40.73–65.91	16.27–81.40	1669.45 (*p* < .001)	99.4%	344.94
Mental health	53.57	46.54–60.61	40.91–69.57	729.26 (*p* < .001)	98.8%	94.38

### SF-36 Physical and Mental Health Summary Measures

**Fig. 2:**
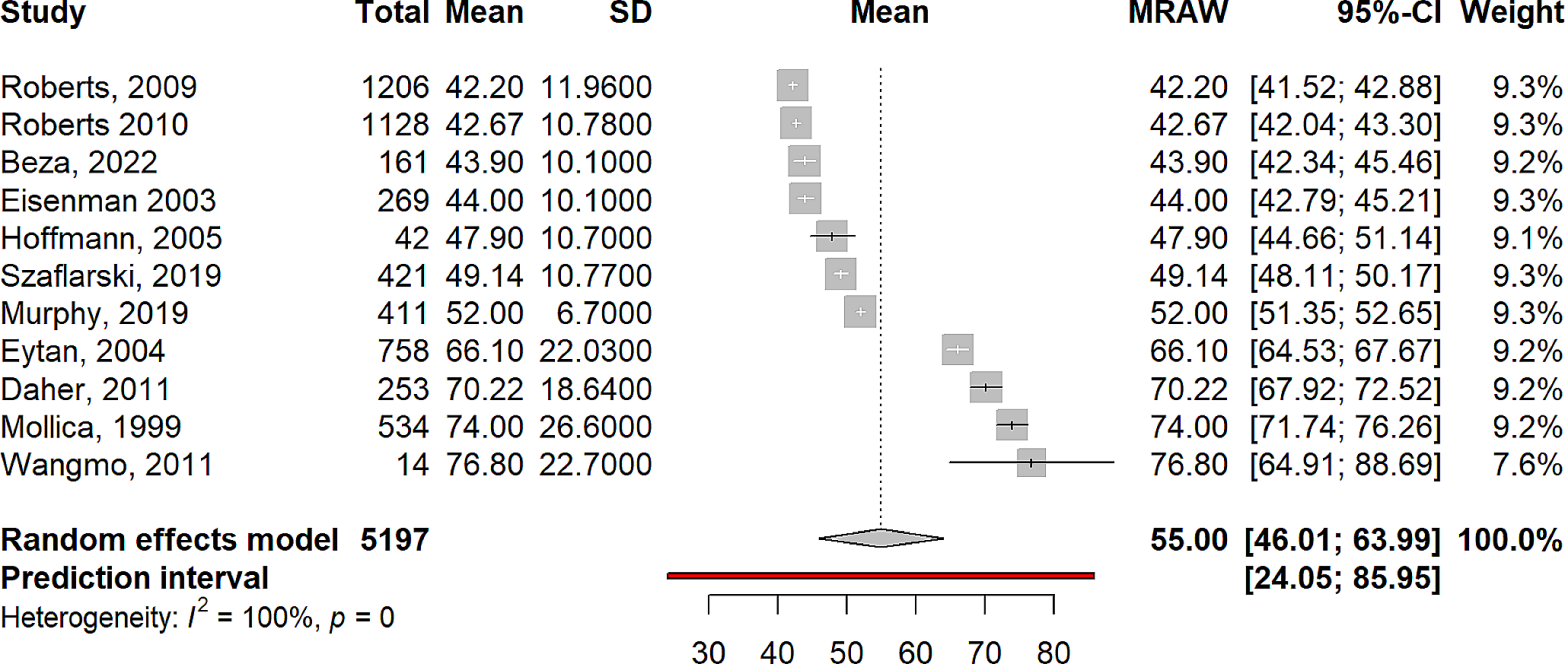
Forest plot of SF-36 physical functioning summary measure

**Fig. 3:**
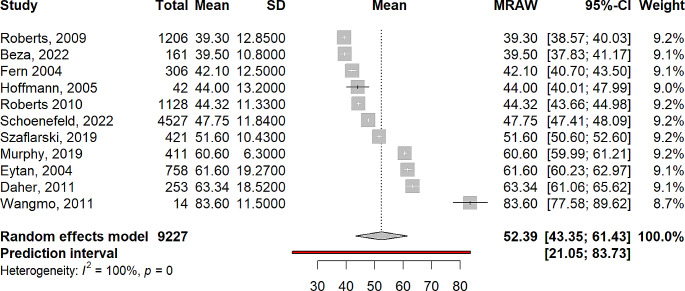
Forest plot of SF-36 mental functioning summary measure

The pooled mean score for the physical summary measure was 54.99 (95% CI 46.01–63.99). Mean scores of individual studies ranged from 42.4 to 76.8. Significant (Q = 2179.44, *p* < .001) and substantial heterogeneity (I^2^ = 99.5%, τ^2^ = 171.29) were observed, meaning study means and standard deviations varied significantly between the studies. Results are summarised in Fig. 2. The pooled mean score for the mental summary measure was 52.39 (95% CI 43.35–61.43). Mean scores of individual studies ranged from 47.75 to 83.6. Significant (Q = 3174.56, *p* < .001) and substantial heterogeneity (I^2^ = 99.7%, τ^2^ = 175.78) were observed, meaning study means and standard deviations varied significantly between the studies. Results are summarised in Fig. 3.

### Sensitivity Analysis

A sensitivity analysis was conducted for each scale and summary measure with outliers removed. Notably scores in studies by Hinton, Sinclair [26] and Wangmo [32] were substantially lower and higher (respectively) than those reported elsewhere. Also excluded were studies above where data was transformed or where data was selectively extracted and those which scored below 70% on quality appraisal instruments. While pooled mean scores shifted slightly with these studies removed, this has little impact on the overall results, with scores still highly heterogeneous. We opted to retain these studies and offer some reflections below on why scores were substantially different in these studies.

### Sub-group Analysis

**Fig. 4:**
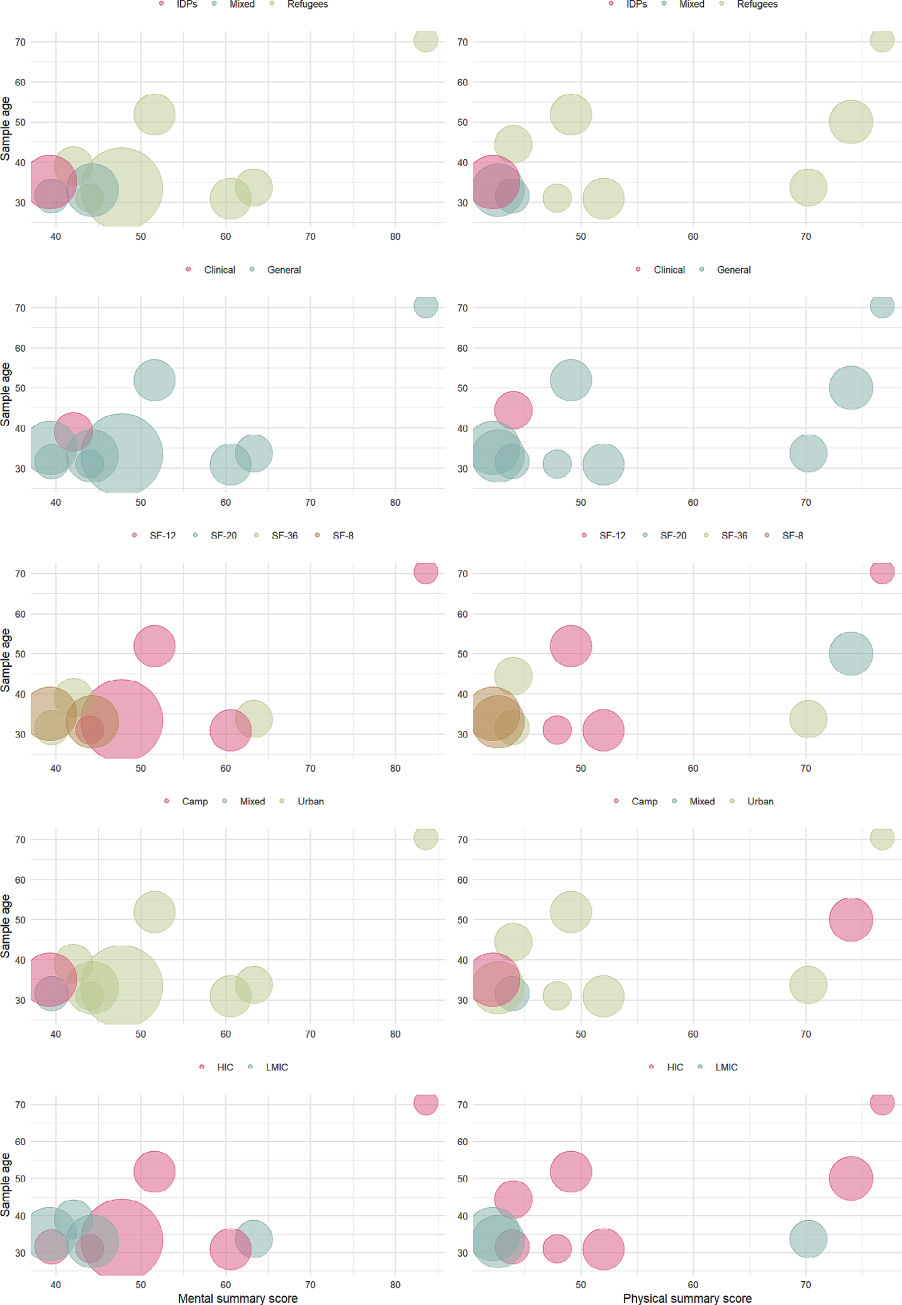
Bubble plots of physical and mental summary measures by mean age of sample. *Note* Bubble size reflects sample size of study. Plots on the left all report mental summary score, while those on the right report physical summary score. Mean sample age is represented on the y axis

While we had planned to run sub-group analyses we opted not to do this prior to undertaking analysis. While 20 studies were included in this meta-analysis, not all reported scores for each scale and summary measure. As noted above the most reported scales were the mental (*n* = 11) and physical (*n* = 11) summary measures. When looking at the studies that reported each of these summary measures, they varied substantially and often we were left with only one study that offered a comparison (for example, only one study was conducted in a clinical (hospital) setting that used both the physical and mental summary measures). Because of this, we did not carry out formal sub-group analysis. We did however speculatively explore the remainder of the studies to see if patterns in the data existed. These results are summarised in Fig. 4. which plots physical and mental summary scores and age, with each colour representing different sample characteristics. There appears to be no obvious relationship between summary scores and age, however there are notably at least three studies that appear to be outliers on both scales. One of these studies had a sample much older than the majority of the other studies, while all studies had samples of refugees and came from general (as opposed to clinical) populations. Notably, some studies also appeared to have very different scores based on study characteristics. For example, two studies carried out in high income countries had at least two outliers, with two studies with substantially higher physical and mental summary scores than others, while the two studies carried out in refugee camps had vastly different physical summary scores. These findings will be discussed below in light of the broader literature.

### Study Quality and Publication bias

To assess the quality of the studies included in this review, the JBI instrument for cross sectional studies was used. The results and rating for each study are included in supplementary material (Table S1). Generally studies scored well, however one persistent issues across a number of studies related to the scoring of the SF-36, where in many cases this was not reported with adequate transparency. Only one study failed to meet 70% of the criteria put forth in the tool [26]. No evidence of potential publication bias was found, based on the funnel plots (Appendix Figures S9 and 10) and a nonsignificant Eggers test (*p* = .11) for the physical summary and (*p* = .80) for the mental summary scales.

## Discussion

This study provides insight into health-related quality of life of refugees and asylum seekers by examining SF-36 scores amongst 18,418 refugees and asylum seekers. Mean scores for the physical and mental summary scales were 54.99 and 52.39 respectively. Sub scales ranged from 49.60 (vitality) to 65.54 (physical functioning). Scores between studies were highly heterogenous on all summary measures and sub-scales, meaning scores varied substantially.

**Fig. 5:**
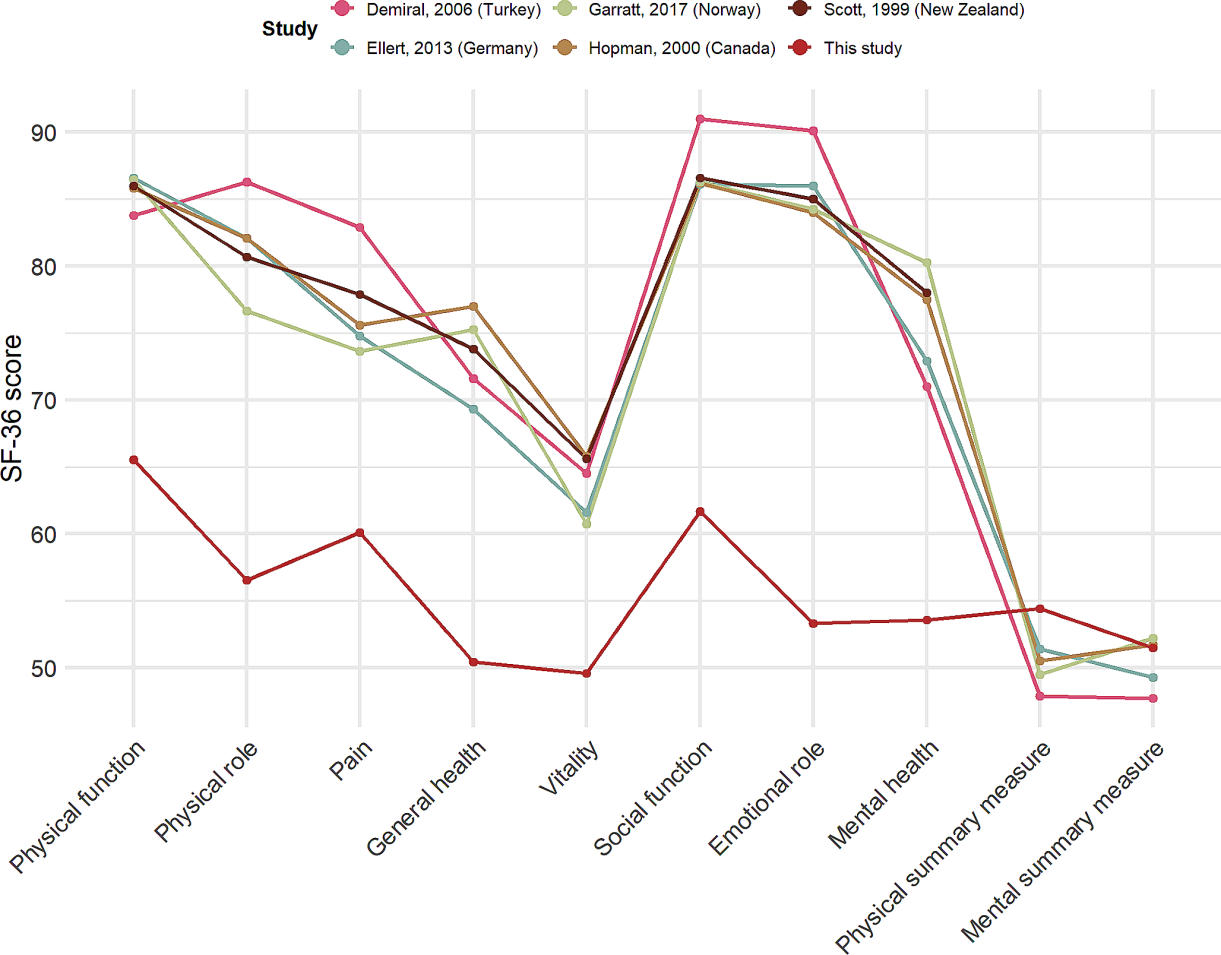
pooled SF-36 scores from this study compared to scores found in other studies

Beyond the differences between studies included here, one advantage of utilising SF-36 scores to explore HRQoL is that it allows for comparisons between different populations. To offer a point of comparison we explored five further studies that employed large general samples from Germany [42], Canada [43], Norway [44], New Zealand [45] and Turkey [46] and compared these with the pooled mean scores found in this study. These results are summarised in Fig. 5. This comparison, that on all scales, the pooled mean scores in this study were lower, except for the physical and mental summary measures where mean scores appear comparable. This suggests that generally, refugees and asylum seekers to have lower SF-36 scores than those in the general population in a number of high and middle income countries.

While we did not opt to carry out formal sub-group analysis it is worth reflecting on some of the outliers we identified in the above scores, along with what insights these might provide given the existing literature. The study by Wangmo [47] is notable in that it sampled a group much older than what was found in other studies. This study examined SF-36 scores amongst elderly Tibetans settled in both Switzerland and India. When extracting data we opted to only extract data from those resettled in Switzerland as the scores between the groups varied substantially, with those in India having much lower physical and mental summary scores. This study begins to provide some insight into why the group resettled in Switzerland had far higher scores, and arguably sheds light on some of the variability seen between many of the studies above. One important factor this study identified was the benefits and support provided in Switzerland, compared to India, such as social security and healthcare. The reasons why the other studies that were outliers on the physical [28] and mental summary scores [34] were not immediately apparent. Mollica [28] examined physical summary scores amongst Bosnian refugees in a camp in Croatia, while Daher [34] examined mental summary scores amongst Iraqi refugees resettled in Malaysia, this study noted that the sample was relatively young, with the vast majority aged between 18 and 29 years old having travelled to Malaysia to both study and escape the political instability in Iraq; this may at least partially explain why scores were higher on this measure for this group. One final study by Hinton, Sinclair [26] where scores were also outliers, examined refugees from Vietnam and Cambodia, resettled in the US with psychiatric diagnoses, in line with the broader literature (see below) this may explain why scores were much lower for this group.

Together these results suggest that refugee quality of life is generally poorer than that of the general population in a number of host countries. What is also apparent is that this can vary quite substantially. One issue that is not well clarified by this review were the factors that determined health-related quality of life. While we did not opt to carry out formal sub-groups analysis, we also did not find any apparent patterns in our data that would suggest study or sample characteristics being predictive of SF-36 scores. Looking at the broader literature however, there are several potential explanations. These include the diversity of the samples, that is, refugees and asylum seekers have vastly different experiences, come from a variety of backgrounds and are exposed to vastly different potentially traumatic experiences. In resettlement, a range of different and varying challenges are faced dependent on the country of resettlement. This is reflected in the broader literature that has explored the factors that impact quality of life amongst refugees and also perhaps explain some of the heterogeneity in this study. In a systematic review that examined 23 papers that explored the factors that were associated with refugee quality of life, van der Boor, Amos [7] found that the most salient factors when it came to quality of life were related to social integration and “having established social networks”. Having a mental health disorder was the most salient factor when it came to reductions in quality of life. When it comes to mental health, several factors, such as isolation have been found to predictive of worse mental health [48]. Studies have also found a significant relationship between factors related to host countries’ refugee policy, whether people are detained and the process by which they can apply for protection and their wellbeing [49]. In practical terms, with the results of this study and the broader literature, this means that in resettlement, while quality of life is generally poorer for refugees, this is not always the case and the factors that may contribute to this are numerous. This study also highlights the difference between difference elements of wellbeing, for example emotional and physical wellbeing, with subs-scale scores varying substantially between studies. These results and the broader literature highlight the need for a comprehensive and individualised exploration of quality of life amongst refugees who are resettled taking into account the range of individual, social, culture and political factors that may influence this.

There are at least two notable limitations worth mentioning in relation to this study. First, it was not always clear how studies scored the SF-36 and in particular how they calculated physical and metal summary scores. This was not always detailed in papers and often studies only included summary scores (and not the scores of each of the sub-scales). While these studies may have been scored correctly, there was often a lack of detail about how this was done. This may explain why the sub scale scores here are lower than most published studies, while the physical and mental summary scores are comparable. There is a need for future studies to offer greater transparency around the scoring of these instruments. Second, and following from what was said above, we opted to include a relatively broad sample of refugees and asylum seekers in this study, all who had vastly different experiences, through fleeing their country of origin to their experiences in resettlement, all having vastly different impacts on quality of life. Future meta-analyses that examine the quality of life of refugees should consider whether it may be of benefit to examine quality of life amongst a more focused sample because of these differences.

This paper adds to discussions in relation to refugee health and in particular refugee health related quality of life. These findings suggest that while refugee health-related quality of life is generally poorer than that found in the majority of countries, when compared to the general population, this can vary substantially. Future research is needed to more systematically explore the factors that influence quality of life in these populations.

## Electronic supplementary material

Below is the link to the electronic supplementary material.

Supplementary Material 1
