# Supplementary material for: Health Related Quality of life Amongst Refugees: A meta Analysis of Studies Using the SF-36
Source: J Immigr Minor Health. 2024 Jul 3;26(5):925–35. doi: 10.1007/s10903-024-01615-4 (PMC11413143; doi:10.1007/s10903-024-01615-4)
Supplement: Supplementary file 1 — Supplementary Material 1 [file 10903_2024_1615_MOESM1_ESM.docx]

Supplementary material

Figure S1. Forest plot of SF-36 physical functioning scale


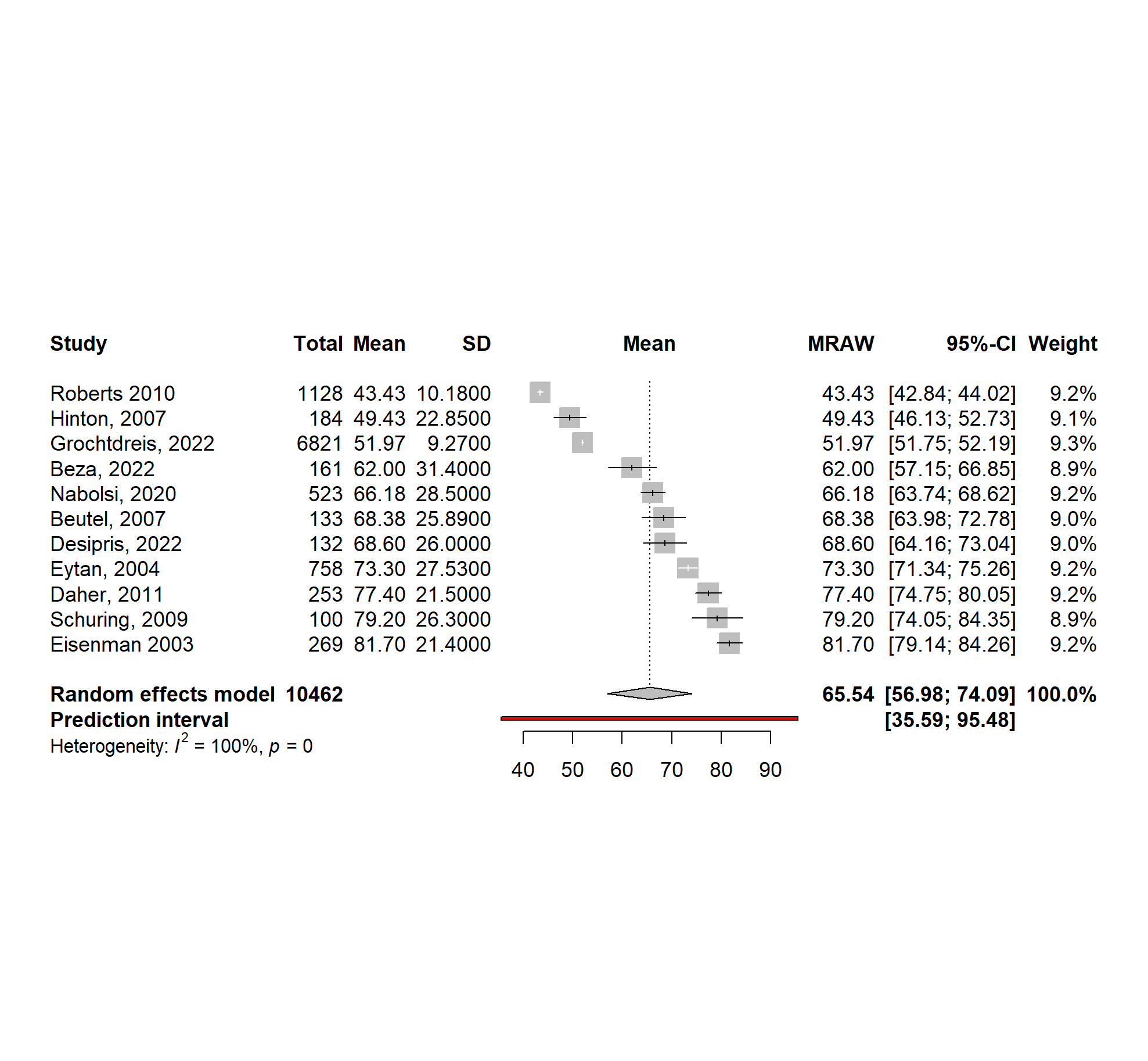


Figure S2. Forest plot of SF-36 physical role scale


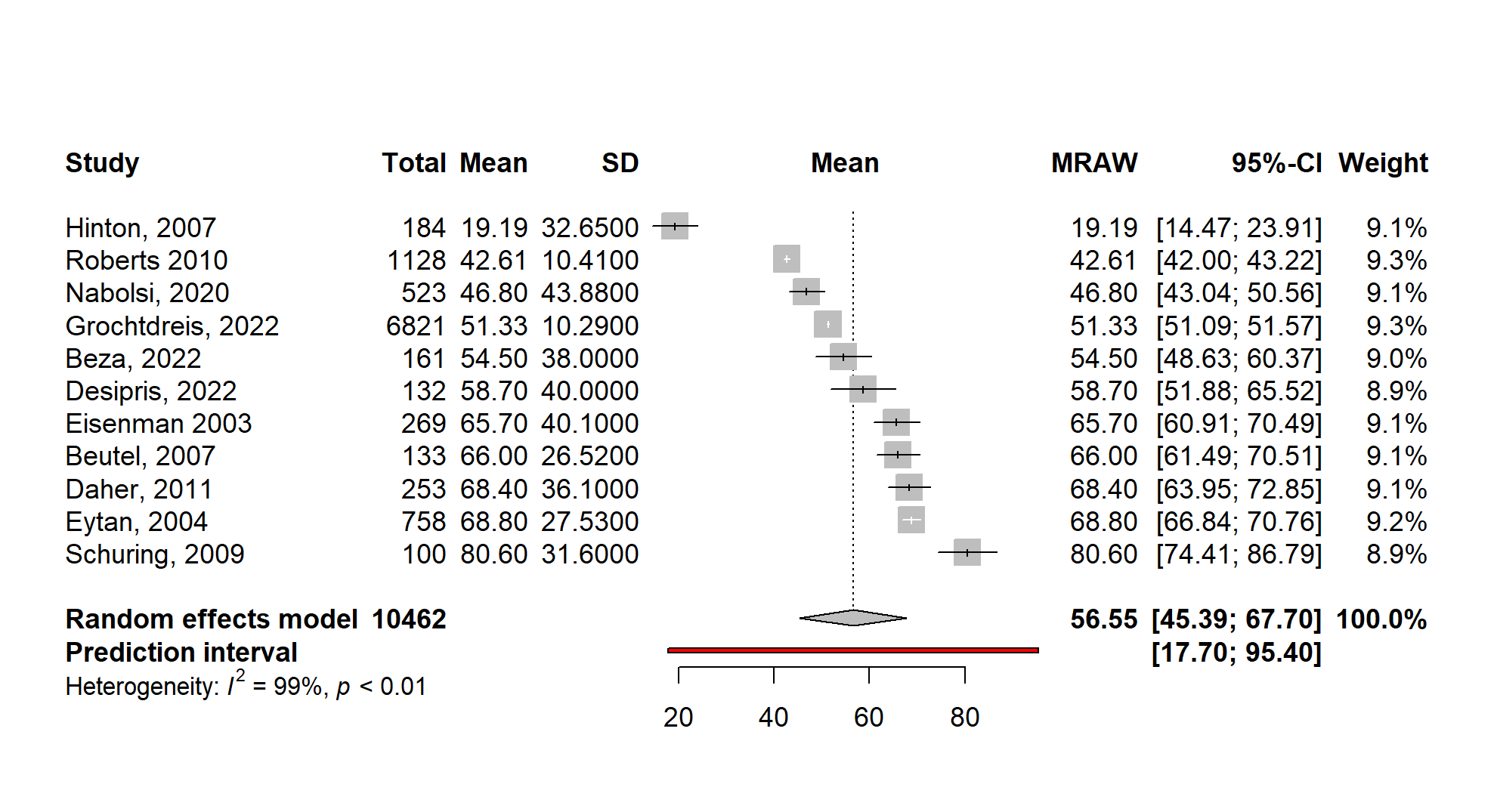


Figure S3. Forest plot of SF-36 pain scale


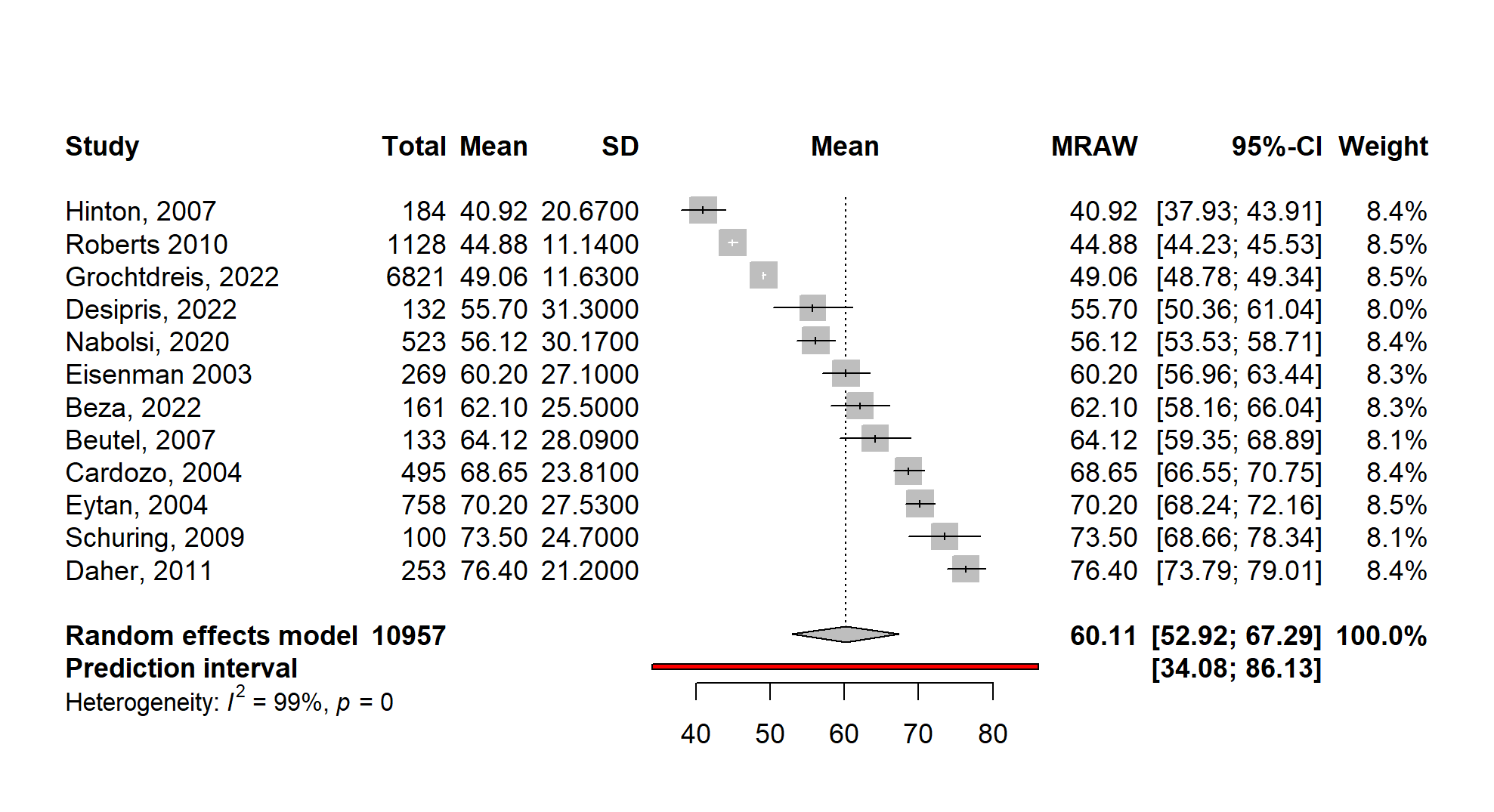


Figure S4. Forest plot of SF-36 general health scale


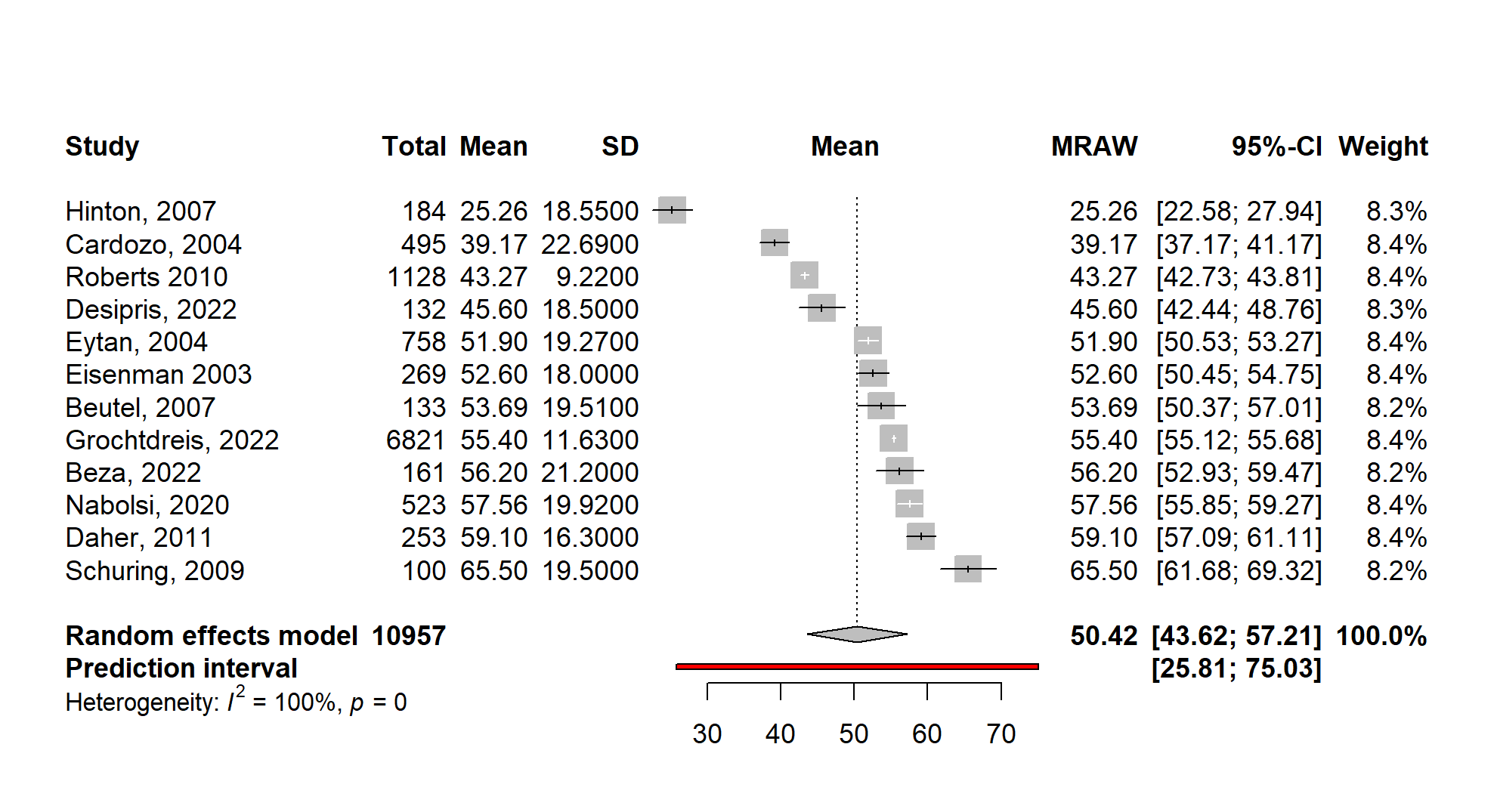


Figure S5. Forest plot of SF-36 vitality scale


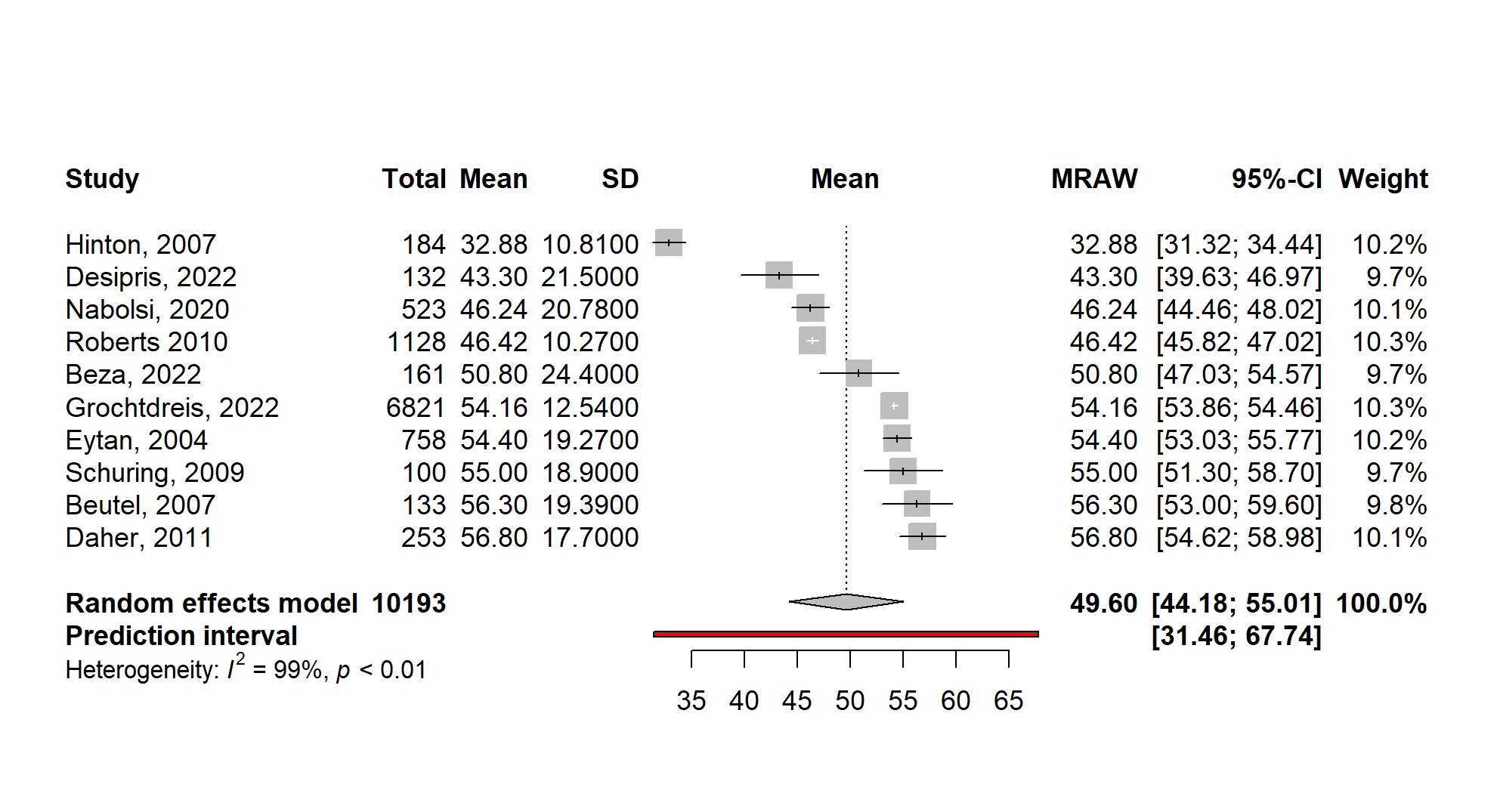


Figure S6. Forest plot of SF-36 social functioning scale


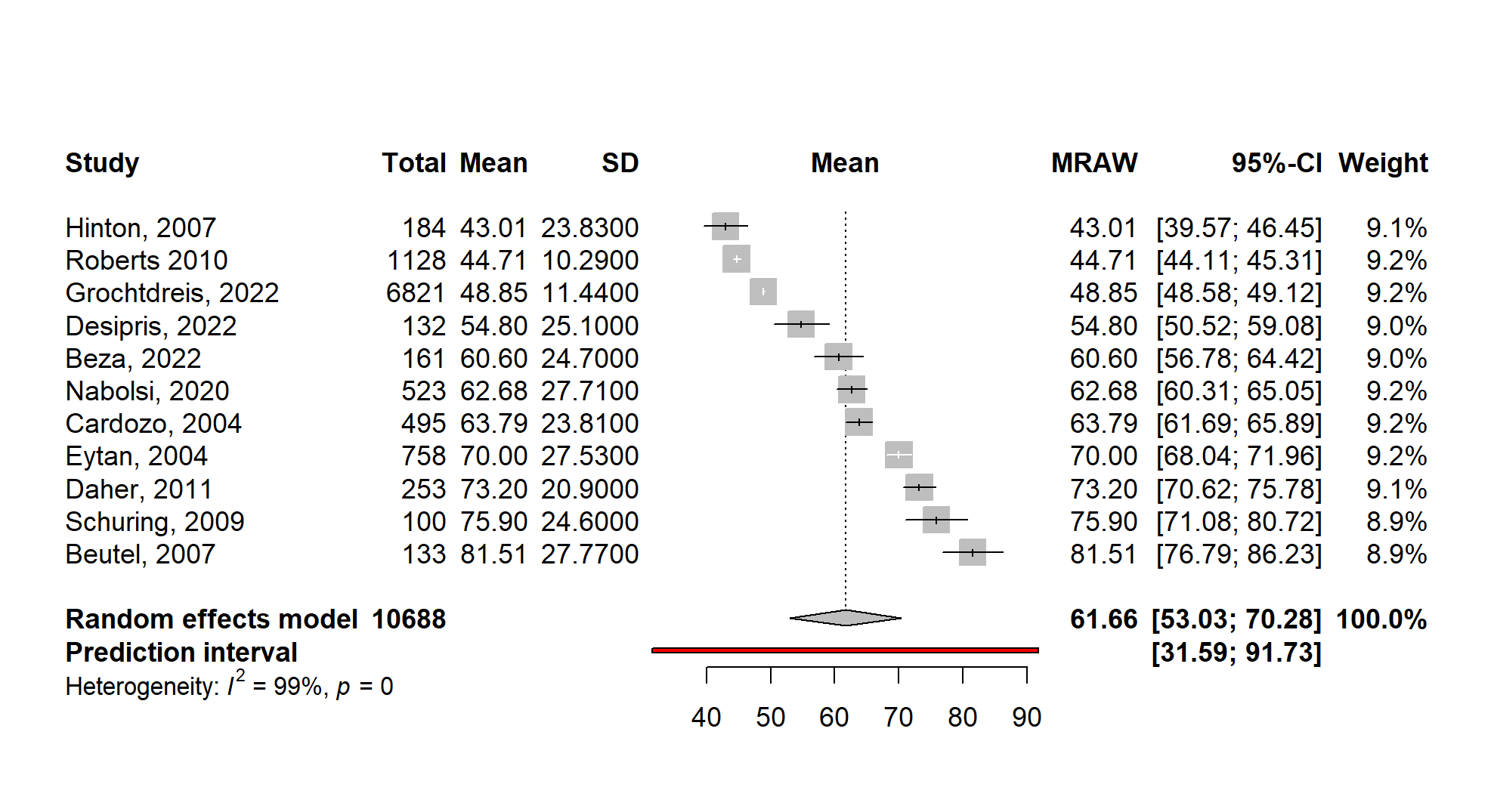


Figure S7. Forest plot of SF-36 emotional role scale


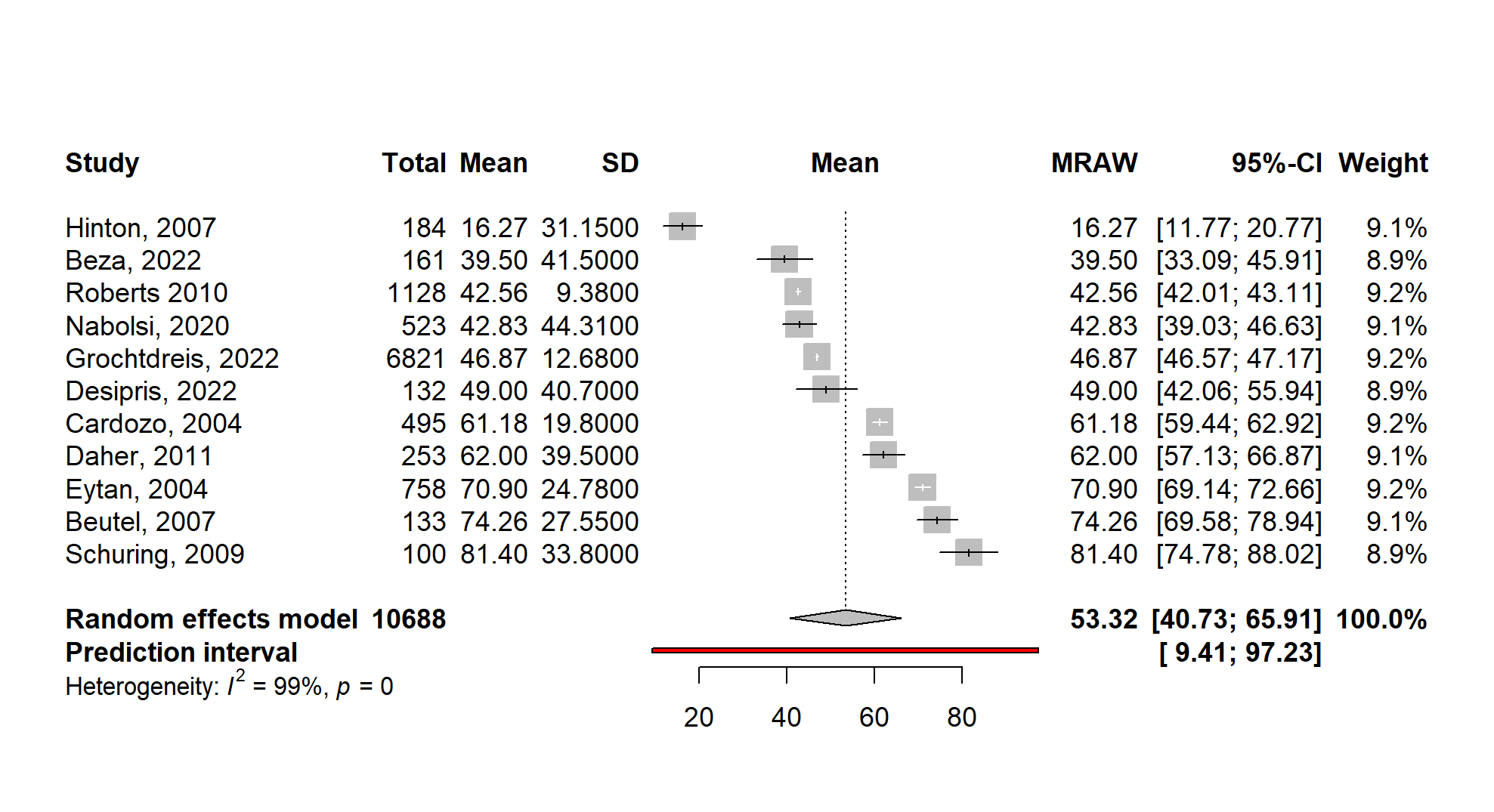


Figure S8. Forest plot of SF-36 mental health scale


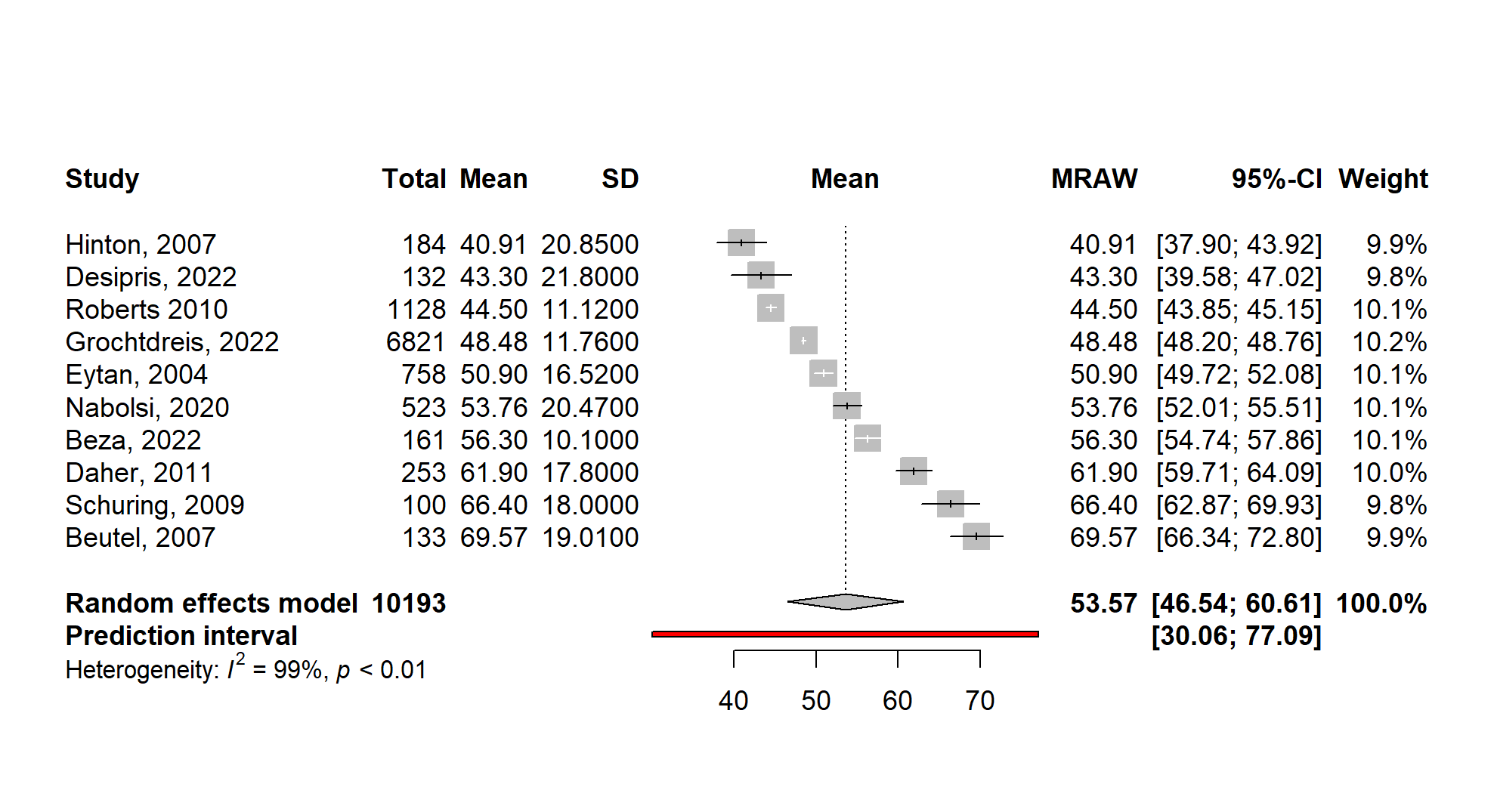


Figure S9. Funnel plot – Physical summary scores


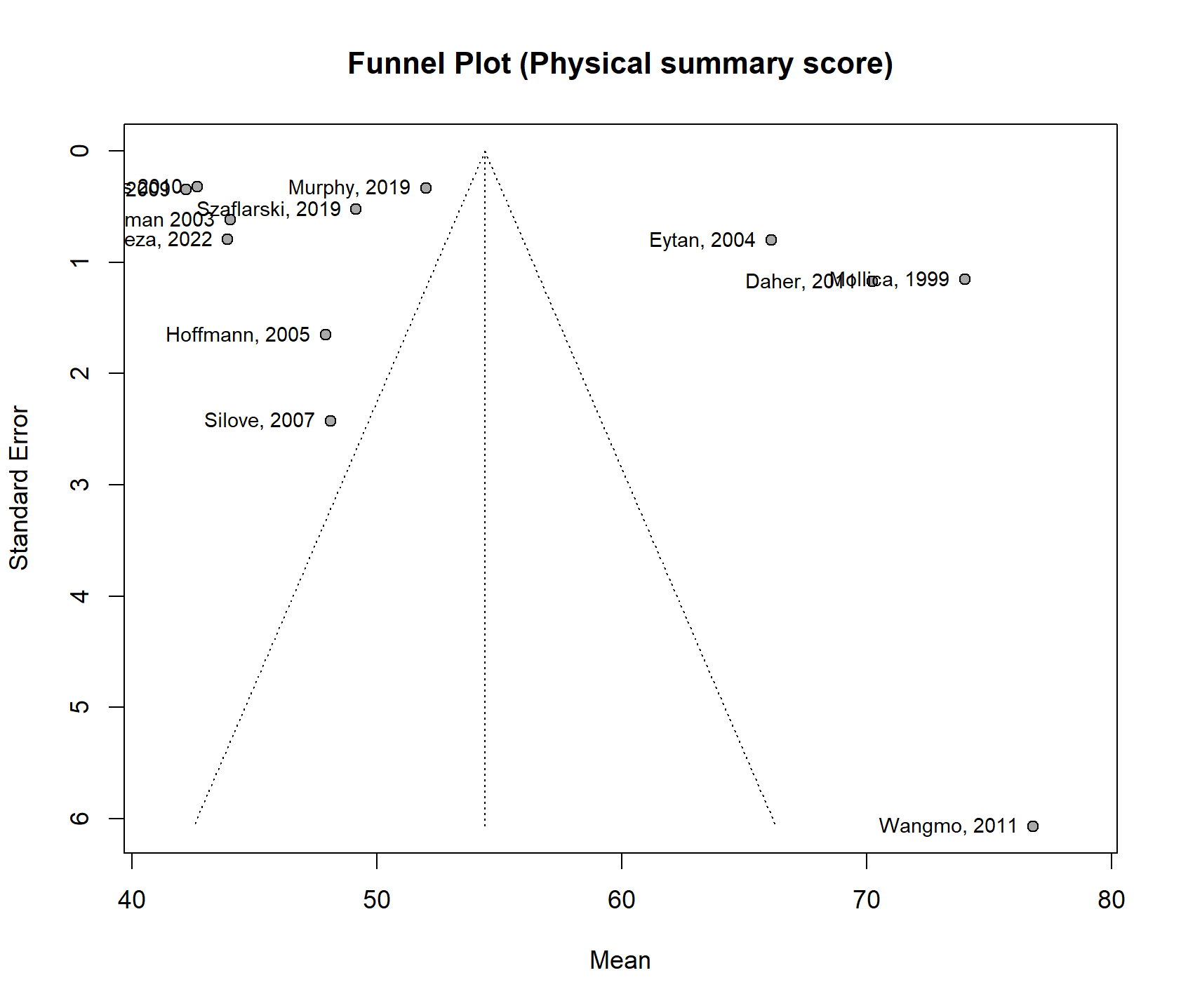


Figure S10. Funnel plot. Mental summary scores


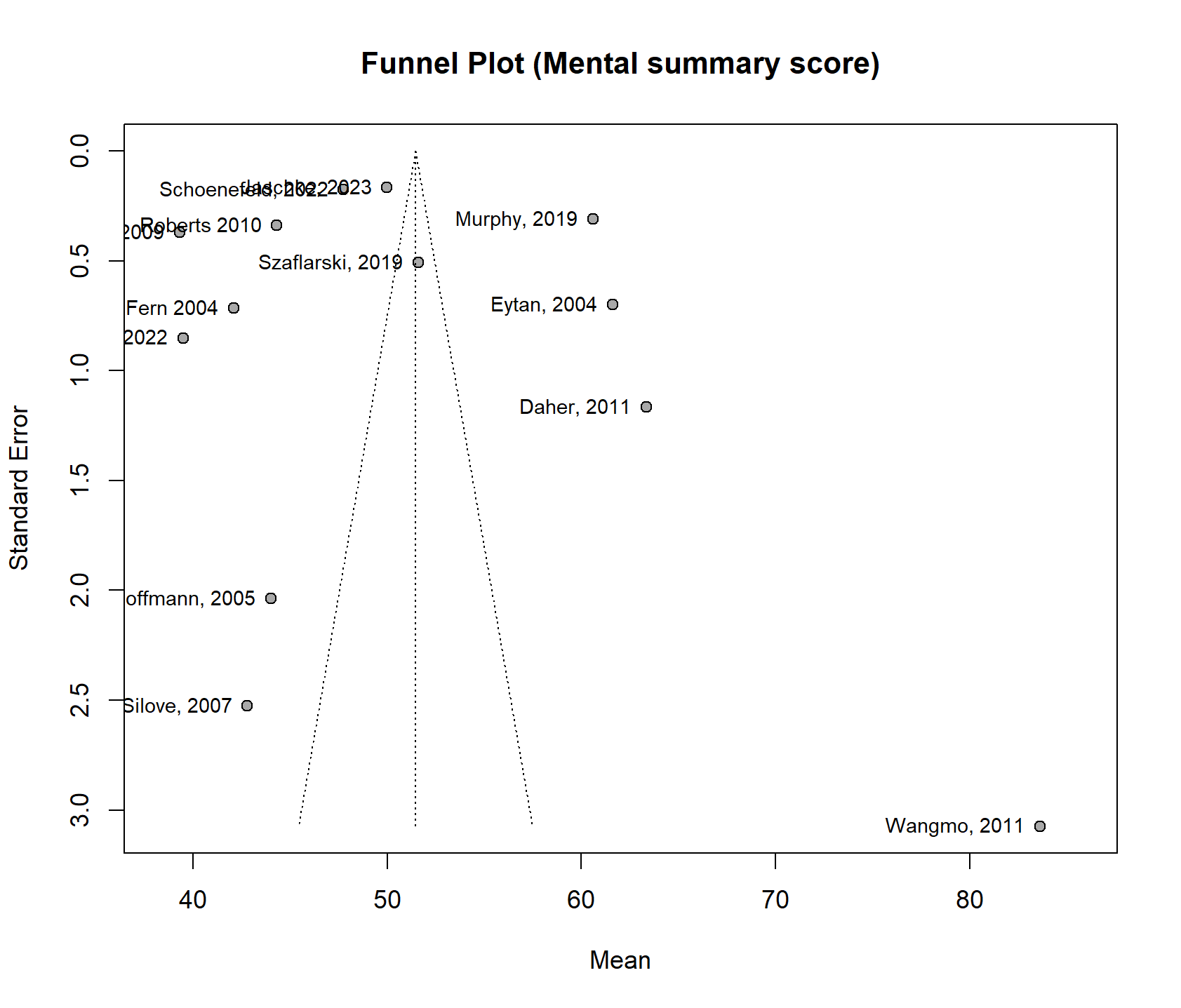


Table S1. Quality appraisal results, utilising JBI cross sectional instrument

| **Author, year** | **1.       Were the criteria for inclusion in the sample clearly defined?** | **2.       Were the study subjects and the setting described in detail?** | **3.       Was the exposure measured in a valid and reliable way?** | **4.       Were objective, standard criteria used for measurement of the condition?** | **5.       Were confounding factors identified?** | **6.       Were strategies to deal with confounding factors stated?** | **7.       Were the outcomes measured in a valid and reliable way?** | **8.       Was appropriate statistical analysis used?** |  |  |  |
| --- | --- | --- | --- | --- | --- | --- | --- | --- | --- | --- | --- |
| Beutel, 2007 | Yes | Yes | Yes | N/A | Yes | Yes | Yes | No |  |  |  |
| Beza, 2022 | Yes | Yes | Yes | N/A | Yes | Yes | Yes | No |  |  |  |
| Cardozo, 2004 | Yes | Yes | Yes | N/A | Yes | Yes | No | Yes |  |  |  |
| Daher, 2011 | No | Yes | Yes | N/A | Yes | Yes | Yes | Yes |  |  |  |
| Desipris, 2022 | Yes | Yes | Yes | N/A | Yes | Yes | Yes | No |  |  |  |
| Eisenman 2003 | Yes | Yes | Yes | N/A | Yes | Yes | No | No |  |  |  |
| Eytan, 2004 | Yes | Yes | Yes | N/A | Yes | Yes | Yes | No |  |  |  |
| Fern 2004 | Yes | Yes | Yes | N/A | Yes | Yes | No | Yes |  |  |  |
| Grochtdreis, 2022 | Yes | Yes | Yes | N/A | Yes | Yes | No | Yes |  |  |  |
| Hinton, 2007 | Yes | No | Yes | N/A | N/A | N/A | Yes | Yes |  |  |  |
| Hoffmann, 2005 | No | Yes | Yes | N/A | No | No | Yes | No |  |  |  |
| Mollica, 1999 | Yes | Yes | Yes | N/A | Yes | Yes | Yes | No |  |  |  |
| Murphy, 2019 | Yes | Yes | Yes | N/A | Yes | Yes | No | Yes |  |  |  |
| Nabolsi, 2020 | Yes | Yes | Yes | N/A | Yes | Yes | Yes | Yes |  |  |  |
| Roberts 2010 | Yes | Yes | Yes | N/A | Yes | Yes | Yes | Yes |  |  |  |
| Roberts, 2009 | Yes | Yes | Yes | N/A | Yes | Yes | Yes | Yes |  |  |  |
| Schonfeld, 2022 | Yes | Yes | yes | n/a | Yes | Yes | yes | No |  |  |  |
| Schuring, 2009 | Yes | Yes | Yes | N/A | Yes | Yes | No | No |  |  |  |
| Szaflarski, 2019 | Yes | Yes | Yes | N/A | Yes | Yes | Yes | No |  |  |  |
| Wangmo, 2011 | Yes | Yes | Yes | N/A | Yes | No | Yes | No |  |  |  |
